# Nature of the Electrical Double Layer on Suspended Graphene Electrodes

**DOI:** 10.1021/jacs.2c03344

**Published:** 2022-07-18

**Authors:** Shanshan Yang, Xiao Zhao, Yi-Hsien Lu, Edward S. Barnard, Peidong Yang, Artem Baskin, John W. Lawson, David Prendergast, Miquel Salmeron

**Affiliations:** † Materials Sciences Division, Lawrence Berkeley National Laboratory, Berkeley, California 94720, United States; ‡ Department of Chemistry, University of California-Berkeley, Berkeley, California 94720, United States; § Department of Materials Science and Engineering, University of California, Berkeley, California 94720, United States; ∥ Molecular Foundry, Lawrence Berkeley National Laboratory, Berkeley, California 94720, United States; ⊥ NASA Ames Research Center, Moffett Field, California 94035, United States

## Abstract

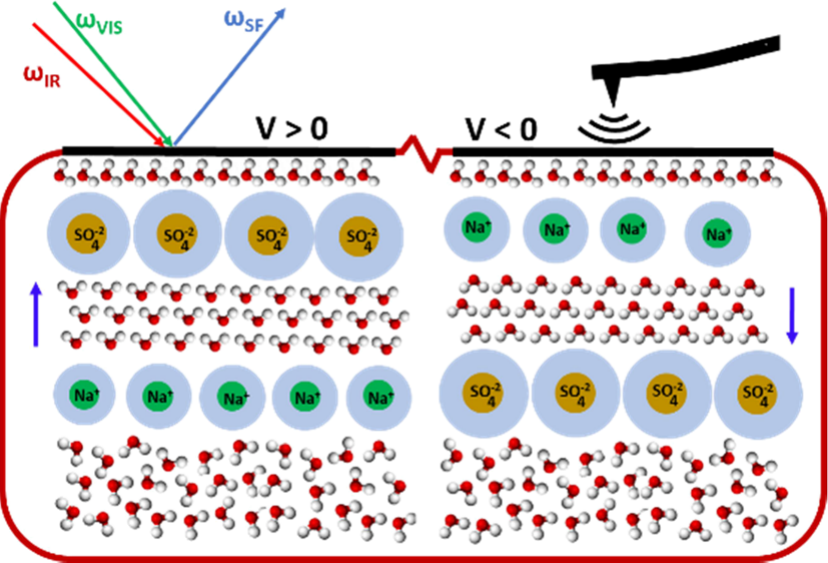

The structure of interfacial water near suspended graphene electrodes in contact with aqueous solutions of Na_2_SO_4_, NH_4_Cl, and (NH_4_)_2_SO_4_ has been studied using confocal Raman spectroscopy, sum frequency vibrational spectroscopy, and Kelvin probe force microscopy. SO_4_^2–^ anions were found to preferentially accumulate near the interface at an open circuit potential (OCP), creating an electrical field that orients water molecules below the interface, as revealed by the increased intensity of the O–H stretching peak of H-bonded water. No such increase is observed with NH_4_Cl at the OCP. The intensity of the dangling O–H bond stretching peak however remains largely unchanged. The degree of orientation of the water molecules as well as the electrical double layer strength increased further when positive voltages are applied. Negative voltages on the other hand produced only small changes in the intensity of the H-bonded water peaks but affected the intensity and frequency of dangling O–H bond peaks. The TOC figure is an oversimplified representation of the system in this work.

## Introduction

The graphene–electrolyte interface plays an important role in many applications and technological fields, such as electrocatalysis, energy storage, water desalination,^1,2^ electricity generation,^3−6^ and environmental^7−14^ and biological sensors.^15^ In most of these applications, graphene is in contact with the electrolyte and with a supporting substrate. To improve our understanding of the electrical double layer (EDL) at the graphene–electrolyte interface without interference from the substrate, we used suspended graphene, which also separates the solution from the ambient air.

Graphene is normally assumed to be hydrophobic,^16^ with a specific affinity for cations due to their interactions with graphene defects and π-orbitals.^17,18^ In recent work, an affinity for anions, including OH^–^, Cl^–^, and SO_4_^2–^, has been proposed.^19−21^ The ions are the main factors in the formation of the EDL, which is strongly influenced by the differential segregation of the electrolyte ions to the interface, driven by various physical and chemical forces. One arises from the image charge, where the abrupt change of dielectric properties across the solution–electrode interface leads to polarization effects that can be described by image charges outside the solution. This effect however does not lead to differential segregation here, as both cations and ions are affected alike. However, image charge interactions may assist the ion segregation at the interface in the presence of other mechanisms that break the symmetry between anions and cations. Another is the segregation of ions out of the solution due to the interplay of enthalpic and entropic forces in ion solvation^22,23^ and by the different disruption of the H-bonding structure of water near ions in the bulk and interface. A third driver is the formation of bonds between ions and the electrode, of covalent, ionic, or van der Waals character. Finally, external forces such as the applied bias also play a crucial role. The purpose of this work is to obtain a molecular level understanding of these driving forces and the structure of the electric double layer they create. As we show below, the combination of Raman spectroscopy, sum frequency vibrational spectroscopy (SFVS), and Kelvin probe force microscopy (KPFM), together with the use of suspended graphene electrodes, provides a unique perspective and insight for such studies.

Three salts, Na_2_SO_4_, (NH_4_)_2_SO_4_, and NH_4_Cl, were chosen because of their distinctive adsorption behavior at the air/water interface.^24^ Through Raman measurements, we found that the doping of graphene by electrolyte species is very small at bulk concentrations below 10 mM and thus can be ignored. Under an open circuit potential and for positive voltages, SO_4_^2–^ ions are the dominant species at the interface. Spectra acquired at bias voltages on each side of the charge neutral point (CNP), also called the point of zero charge, showed an asymmetric change with voltage for H-bonded bulk water molecules and for molecules with dangling OH groups at the interface. These changes are also evident in KPFM measurements of the contact potential difference (CPD) between the tip and graphene. Such results are partially consistent with previous observations.^9,21^ The dangling O–H stretch vibrational peak of water is largely unaffected by changes in concentration and applied bias, except at voltages below −0.3 V, where it shifts to a lower frequency and gets buried into the peaks of H-bonded water, implying a change of hydrophilicity at a negatively biased graphene electrode.

## Results and Discussion

### Hydrophobicity of Graphene and Contamination Effects

It has been reported that graphene may suffer from air-borne hydrocarbon contamination.^25−28^ To check the effects of contamination, we prepared graphene in three different ways before transferring to the water surface: (1) ultraviolet (UV) irradiation for 5 min, (2) washing in a mixture of acetone and isopropyl alcohol (IPA) (1:3) for 30 min, and (3) as received, i.e., no treatment (see Section S3). To assess the amount of hydrocarbon contamination on graphene, we measured the intensity of the C–C and C–H stretch and CH_2_ twist peaks with Raman spectroscopy, as described in Section S3. With reference to the peaks from a saturation layer of polyethylene formed by immersing graphene in a 1.6 μM polyethylene/CCl_4_ solution, we found that the level of contamination was 0.05 ML after a UV treatment, 0.13 ML after IPA washing, and 0.14 ML in the untreated sample. However, the “cleanliness” of graphene after these treatments was found to be short lived. As shown in Section S5, after 30–60 min in air in our laboratory conditions following the cleaning procedure, the sample became contaminated again to their precleaning level. Therefore, all experiments reported here were performed on samples with a hydrocarbon coverage of about 0.14 ML.

### Charge State of Graphene from Doping by Solution Ions

**Figure 1:**
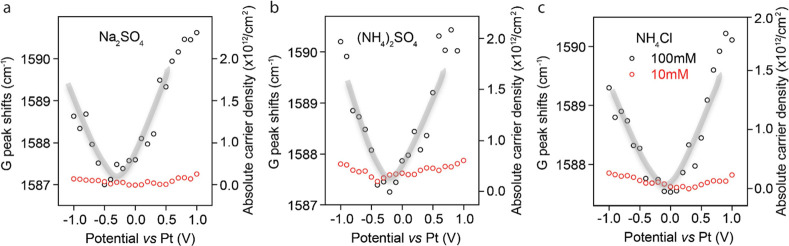
Graphene G peak shift (left *Y*-axis) in Raman spectroscopy, and absolute carrier density (right *Y*-axis), for (a) Na_2_SO_4_, (b) (NH_4_)_2_SO_4_, and (c) NH_4_Cl as a function of bias relative to the Pt electrode. The gray lines are visual guides. Two concentrations, 10 mM (red data points) and 100 mM (black), are shown for each salt. Charging from doping was found to be negligible for salt concentrations of 10 mM and below but important at higher concentrations. The graphs show that the neutral (no charge) point is between 0.0 and −0.5 V for these salts.

As shown in Figure 1, the graphene electrode can be doped by the ions in the solution near the interface. This charge was measured from the G-peak frequency shift in the Raman spectra, as shown in Figure 1. As can be seen, for a concentration of 100 mM, the doping effect is very clear and reaches a minimum vs bias that corresponds to the CNP, also known as the point of zero charge, between 0.0 and −0.5 V bias, where the charge doping on graphene is near zero. As the salt concentration decreases, the doping of graphene decreases also, becoming negligible at 10 mM concentration (red data points) for all bias voltages.

### Effect of Salt Concentration on the Double Layer Field

The differential segregation of cations and anions near the graphene–water interface creates an electric field in the double layer with an intensity that depends on ion concentration. The field affects the vibration spectrum of the water molecules and changes the intensity of the peaks in the O–H stretch region, between 3000 and 3600 cm^–1^. This change is the result of the symmetry rules governing the sum frequency generation (SFG) process, which requires a lack of inversion symmetry. At the interface, there is an intrinsic lack of inversion symmetry manifested by the intense peak around 3620 cm^–1^ due to the dangling O–H stretch vibration mode of the water molecules at the interface next to graphene, which is not affected by the salt concentration. However, most of the H-bonded water molecules below the first layer are randomly oriented; i.e., they are symmetric as an ensemble within a volume of wavelength dimensions and therefore cannot generate an SFG output. The orientation ordering is not abrupt but decays rapidly from the interface to the bulk interior. As a result, the O–H stretch vibration peaks of the molecules have small intensities.

**Figure 2:**
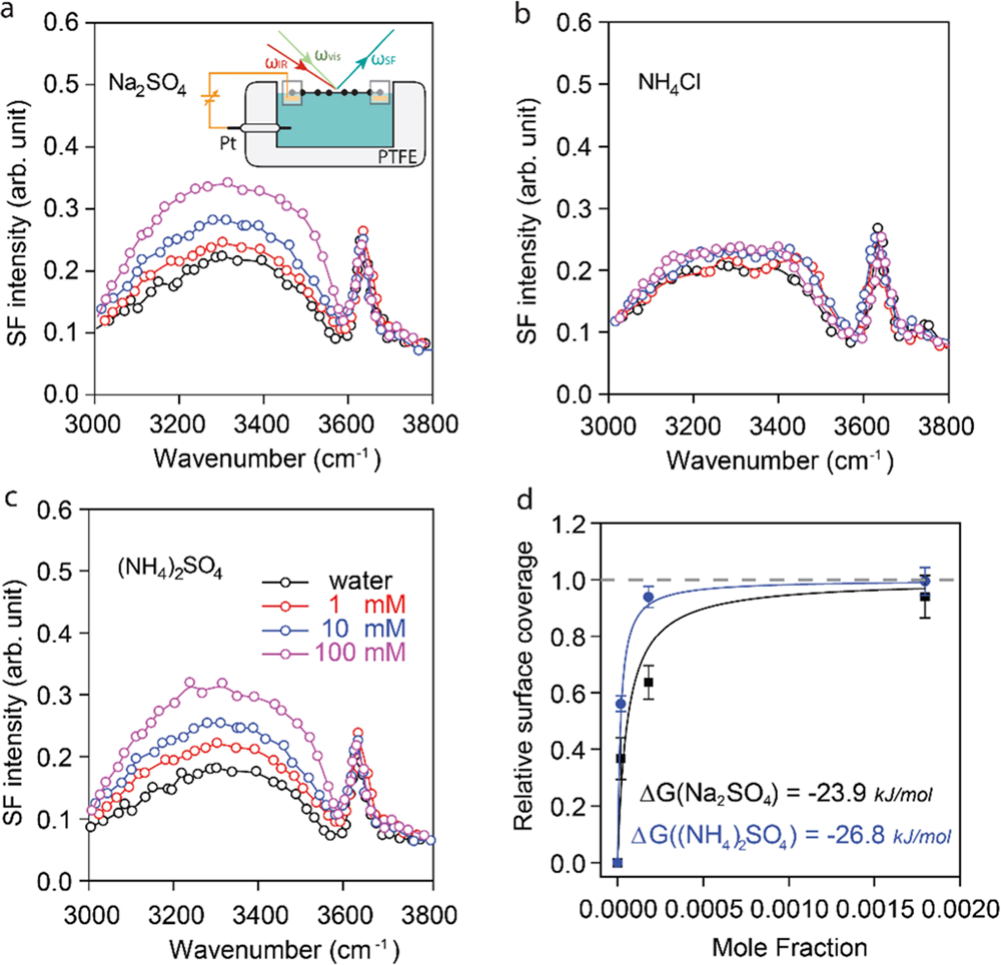
SFVS measurement of the vibrational spectrum of water in the O–H stretch region at an open circuit potential for (a) Na_2_SO_4_, (b) NH_4_Cl, and (c) (NH_4_)_2_SO_4_, for concentrations of 0 mM, (i.e., pure water, black), 1 mM (red), 10 mM (blue), and 100 mM (magenta), respectively. The region between 3000 and 3600 cm^–1^ corresponds to the H-bonded water, while the peak at 3650 cm^–1^ is due to the dangling O–H bond of water at the interface. The inset in (a) shows a schematic view of the PTFE (Teflon) electrochemical cell used in the SFVS measurements. The edges of the copper frame holding the suspended graphene are protected by the poly(methyl methacrylate) (PMMA) polymer (translucent square). A wire connected to the Cu frame grounds the sample and allows biasing the graphene with respect to the Pt electrode. The arrows labeled ω_IR_, ω_vis_, and ω_SF_ indicate the infrared (IR), visible, and output SF beams, respectively. (d) Sulfate ion adsorption isotherms from Na_2_SO_4_ (black) and from (NH_4_)_2_SO_4_ (blue) extracted from the intensity of the H-bonded peaks vs concentration as explained in the main text.

We should note here that the probing depth of SFG is not determined by the penetration depth of the photons, which is macroscopic for the visible beam and of micrometers for the IR beam, much larger than the Debye length (of an order of 1 nm), or the Gouy-Chapman length (angstroms). Instead, it is determined by the depth of the illuminated region lacking inversion symmetry. The presence of electric fields tends to orient the molecular dipoles along the field direction, i.e., perpendicular to the interface, which break the inversion symmetry and cause the intensity increase observed in the SFVS signal seen in Figure 2. The fact that the intensity of the H-bonded water peak increases with the sulfate salt concentration indicates a higher density of ions near the interface at an open circuit potential (OCP).

The intensity of the SFVS peaks is determined by the effective surface nonlinear susceptibility, χ_S,eff_^2^ (ω), of interfacial water, which depends on the electric field *E*_DC_ following the expression:^29^
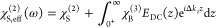
1where χ_S_^(2)^ denotes the contribution from water molecules right at the interface, while the integral describes the contribution from field-induced polarization of water molecules in the diffuse layer. *E*_DC_(*z*) is the distance-dependent field along the surface normal, χ_B_^(3)^ is the third order nonlinear susceptibility of bulk water, and Δ*k*_z_ is the phase mismatch in the SFVS process. The change of χ_S,eff_^2^ (ω) directly reflects the change of *E*_DC_(*z*) in both magnitude and direction.

Figure 2a–c shows the SFVS results for the three salts, with concentrations ranging from 0 to 100 mM at the OCP, which we know is around 0.0 volts relative to the Pt counter electrode. As we see, within measurement error, the spectrum of the graphene/NH_4_Cl interface shows only a small change with the salt concentration (Figure 2b), indicating that NH^4+^ and Cl^–^ ions do not adsorb or segregate differentially to the interface unless, as we show below, they are separated by an externally applied bias. For the two sulfate salts, however, the intensity of the H-bonded water peaks in the 3000–3600 cm^–1^ region increases with the salt concentration. From the contact potential measurements shown below, we know that SO_4_^2–^ ions are preferentially concentrated near the graphene interface at the OCP. The surface coverage of sulfate ions can be deduced from the increase in H-bonded peak intensity relative to that of pure water in Figure 2a,c. According to eq 1, the intensity is correlated with the electric field produced by the surface charge, i.e., ion concentration, which we used to plot the two adsorption isotherms shown in Figure 2d. The details of the calculation are shown in Section S6. From fitting the data with Langmuir adsorption isotherms, we obtained the free energy of segregated sulfate ions, with values of −23.9 and −26.8 kJ/mol, for Na_2_SO_4_ and (NH_4_)_2_SO_4_, respectively, with the 2.9 kJ/mol variance likely being due to the effect of the different positive ions in the salts.

The nature of the driving force responsible for the preferential segregation of sulfate anions is not clear at present. Our experimental measurements indicate that it is not of electrostatic origin since there is no doping charge on graphene at the OCP, as shown by the Raman results in Figure 2. Formation of specific chemical bonds with graphene is also unlikely given the strongly bound water solvation shell around the anions that prevent close proximity for chemical bonding, and van der Waals forces between the anions and graphene are expected to be one order of magnitude smaller than the values obtained. Other factors worthy of consideration include contributions from possible sharing of water molecules between sulfate ions close to the graphene interface, although this is unlikely in view of the large molarity difference between sulfates (<1 M) and water (55 M). Partial desolvation that could introduce a local asymmetry in the solvation molecules around the anion and thus increase their contribution to SFVS is unlikely since the energy of desolvation of the doubly charged sulfate anions is ∼1 eV per molecule.^30−32^ Finally, distortions of the solvation shell of anions near graphene may introduce asymmetries in the vibration modes that could increase the intensity of the SFVS peaks. Since the peak intensities were used to calculate the segregation energy, this could conduce to an overestimation of the energy. These are all important questions that point the way for further investigation in theory and experiments.

### Bias Effects on Ion Adsorption and Water Structure

**Figure 3:**
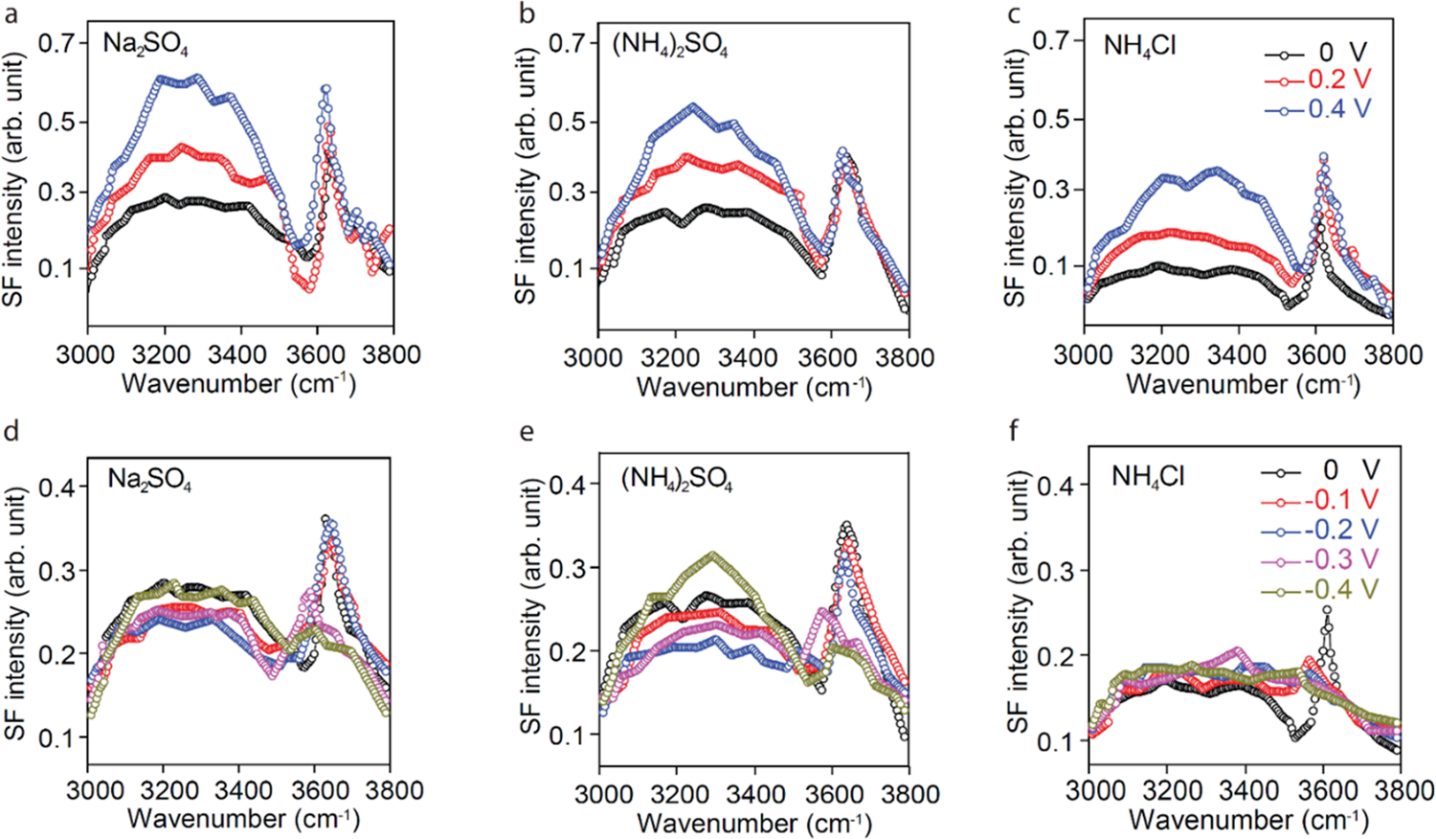
. SFVS as a function of applied bias for 10 mM solutions of Na_2_SO_4_, (NH_4_)_2_SO_4_, and NH_4_Cl. (a, b, c) Top for the positive bias (vs Pt): 0.0 V (black), +0.2 V (red), and +0.4 V (blue). (d, e, f) Bottom for the negative bias: 0 V (black), −0.1 V (red), −0.2 V (blue), −0.3 V (magenta), and −0.4 V (dark yellow).

In the previous section, we showed how increasing the salt concentration at the OCP increases the strength of the EDL field due to differential accumulation of ions at the interface. Here, we present SFVS results showing the changes in the interfacial water structure arising from externally applied fields. A cyclic voltammetry test indicated a capacitive behavior, with the absence of chemical reactions (Figure S7). The results at 10 mM concentration where graphene doping is negligible reveal that the positive bias (Figure 3a–c) increases the intensity of the H-bonded water peaks for all three salts, while the negative bias (Figure 3d–f) decreases the peak intensity to a minimum around −0.2 V for Na_2_SO_4_ and (NH_4_)_2_SO_4_ (Figure 3d–e) and 0.0 V for NH_4_Cl (Figure 3f), indicating that this is the CNP, in agreement with the results of the Raman experiments in Figure 1. Interestingly, however, for negative bias voltages below −0.2 V, a moderate increase in the intensity of the H-bonded water peaks is observed, although barely surpassing the intensity observed in pure water. The different response of anions and cations to the applied bias is another interesting result that is not well understood at present and one that calls for additional experiments and theoretical calculations.

Another important result is the behavior of the water molecules nearest to graphene with a dangling O–H bond peak at 3620 cm^–1^. In studies of the neat water interface with air, it is well known that the dangling O–H stretch peak appears around 3700 cm^–1^ and serves as an indicator of the hydrophobicity of the interface.^33^ The peak does not appear when the interfacial water molecules form H-bonds with a surface, as it occurs on most oxides and hydrophilic interfaces. In our spectra, the peak is found around 3620 cm^–1^. This value can be compared with literature values^34^ where the dangling OD in H_2_O/D_2_O mixtures in contact with graphene is redshifted by about 38 cm^–1^ compared with the air/water interface. The H–D mass difference adds a red-shift of around 50 cm^–1^. In our data, the redshift is 80 cm^–1^, in agreement with the reported values. We have seen that the frequency and intensity of this peak do not change as a function of concentration (Figure 2) nor with the application of a positive bias (Figure 3a–c). However, it does change at the negative bias (Figure 3d–f), decreasing in intensity and red-shifting toward the position of the H-bonded water. This result may indicate that the water molecules next to graphene undergo some orbital hybridization between the dangling H and graphene that causes it to redshift and overlap with the bonded OH region peaks.

### Charge Accumulation near the Graphene Electrode Measured by SFVS and KPFM

**Figure 4:**
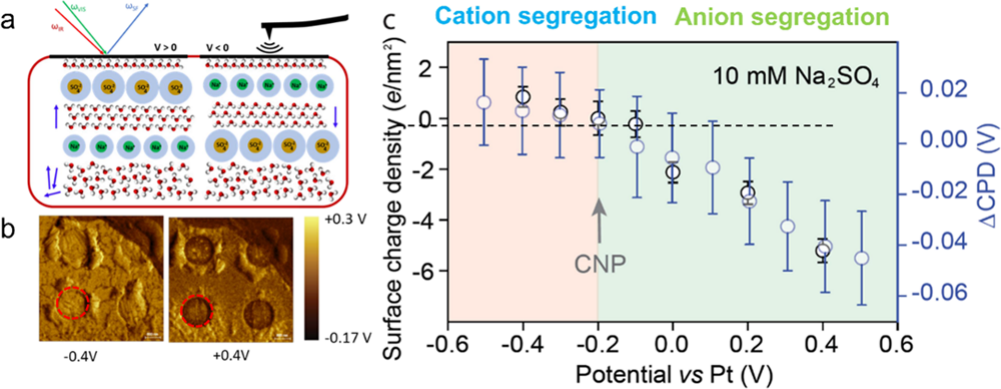
(a) Schematic of the graphene/electrolyte SFVS and KPFM experiments. A very simplified model of the water and ion distribution near the interface is shown. In the model, molecules with dangling O–H bonds are located next to the graphene electrode. This is followed by a layer of solvated anions or cations segregated depending on the polarity, oversimplified here by a single layer of ions, although in reality, the distribution is not abrupt, as shown here. Water in the diffuse region below is partially oriented by the field (highly oversimplified here) created by the segregated ions and their counterions in the diffuse layer. The dipole moment of the field-oriented molecules points on average up or down (arrows on the side), with different degrees for positive and negative polarities relative to the CNP. (b) CPD images from KPFM obtained by scanning the tip over and across suspended graphene in contact with the electrolyte solution. For the KPFM experiment, graphene covered the holes of a gold-coated perforated SiNx membrane. Two images for −0.4 V and +0.4 V bias relative to the Pt counter electrode are shown. The preferential adsorption of SO_4_^2–^ anions is shown by the negative CPD at the positive bias in the suspended graphene region (red circles) relative to the surrounding area. At the negative bias, the CPD is only slightly larger (from Na^+^ accumulation) than that in the surrounding graphene on Au. At the positive bias, the CPD is substantially lower than that in the surrounding area, indicating the higher degree of segregation of SO_4_^2–^ anions. (c) Comparison between the charge density near graphene deduced from the SFVS peak intensity increases of the H-bonded water, (left *Y*-axis, black circle), and the difference in CPD measured by KPFM between suspended graphene and surrounding supported graphene (ΔCPD, blue circle), which is proportional to the ionic charge near the graphene electrode (right *Y*-axis). The pink background denotes positive ion adsorption, and the green background denotes negative ion adsorption. The arrow marks the CNP. Notice the asymmetric surface charging indicating preferential anion adsorption compared to cation adsorption.

The differential segregation of ions to the graphene electrode is also manifested in the increasing ionic charge near the graphene calculated from contact potential change measured by KPFM. For the KPFM measurements, we used a different cell where graphene covered a gold-coated 100 nm thick SiNx membrane^35^ perforated with 1 μm diameter holes (Figure 4). Graphene over the hole regions was suspended and in contact with the solution underneath. Figure 4b shows two KPFM images at −0.4 and +0.4 V bias for a 10 mM Na_2_SO_4_ solution (more images shown in Figure S6). The ionic charge near the graphene electrode that orients the hydrogen bonded water vs bias deduced from the SFVS measurements, using formulas S1–S6 in the SI, is shown in the left *Y*-axis in Figure 4c. The charge from these ions is also proportional to the CPD value measured by KPFM with the tip over the holes minus the CPD value with the tip in the region between holes (ΔCPD). The difference eliminates possible changes in the tip work function due to contamination. The results are plotted in the Figure 4c right *Y*-axis, normalized to match the SFVS charge value at the CNP. As can be seen, the ion charge density curves measured from SFVS and from CPD show the same behavior, with a smaller slope for negative biases (pink background) than for positive biases (green background). Such an asymmetric behavior was also observed on supported graphene electrode/pure water interfaces.^36^ The nonlinear response of the water orientation to different gating potentials and ion species indicates again that the Stern–Gouy Chapman model does not describe properly the EDL at the microscopic scale^37^ and that more advanced EDL models that include effects of solvent dipoles,^38,39^ ion solvation structure,^40^ ion finite size,^41^ and nonelectrostatic forces between molecular species and electrode surfaces^37,42^ should be used. Since the alignment of the water dipoles by the electric field is the result of competition between the torque on the water molecules by the field–dipole interaction and the hydrogen bonding network near the interface, the electric field *E*_DC_ in eq 1, its dependence on distance to the interface, solute type, and ion species should be considered more carefully and needs correction when deducing the surface change density at the interface. However, we believe that the model still provides a reasonable approximation of the field created by the segregated ions, as shown by the good agreement between the charge concentration measured from the increase in SFSV peak intensity and the values obtained using the CPD produced on the graphene as measured by the tip located outside the solution in the KPFM experiments.

## Summary

In summary, through the combined use of Raman spectroscopy, SFVS, and KPFM, we determined the effects of the differential ion segregation at the graphene–electrolyte interface in three salt solutions, Na_2_SO_4_, (NH_4_)_2_SO_4_, and NH_4_Cl, and their effect in creating a double layer structure that orients the interfacial water. The first water layer in contact with graphene has a dangling O–H bond that points to graphene and remains unchanged both with salt concentration and with increasing positive potentials but undergoes a chemical interaction with graphene at negative values that decreases its peak intensity and redshifts its frequency. This indicates that graphene has a hydrophobic character for zero or positive bias, as manifested by the strong dangling O−H bond intensity of the interfacial water molecules, and a hydrophilic character at negative bias, as manifested by the peak frequency shift and the decrease of its intensity. We showed that a preferential anion accumulation at the interface is driven by segregation from the solution bulk, which increased with ion concentration. We have shown that the differential segregation at the OCP is not driven by electrostatic effects nor by formation of specific chemical bonds, which is impeded by the large energy required to desolvate sulfate anions. While the origin of this phenomenon remains unclear, we speculate that it could be due to reorganization of water in the solvation shell that breaks the symmetry of the bonded O–H stretch modes. This therefore requires further experimental or theoretical study of the structure of the EDL. We showed also how externally applied fields further enhance the segregation effects and lead to an increased orientation of interfacial water. The asymmetric change of the field-induced H-bonded water orientation is proved by both SFVS and KPFM and brings to the fore the need for further studies, particularly theory to better understand it. Finally, we found that the dangling O–H peak of the water molecules next to graphene remains largely unchanged as a function of concentration and also under a positive bias but redshifts and decreases in intensity at a negative bias, pointing to orbital hybridization between dangling H and graphene.

## Experimental Method

We used suspended graphene electrodes made from CVD-grown graphene on copper foil (Graphenea Inc). PMMA was first pasted around the edges of the copper foil on both sides. The copper in the middle region was then etched away with a 0.1 M Na_2_S_2_O_8_ solution leaving suspended graphene with a PMMA/copper frame, floating on the water solution. A potentiostat was connected to graphene through the supporting copper frame. To make the sample more robust, two layers of graphene were transferred sequentially, as described in the SI.

In our SFVS-electrochemical cell set-up, we used a picosecond laser system to generate a 1064 nm near-infrared light with a repetition rate of 20 Hz.^43^ A Laser Vision optical parametric generator and amplifier system converts the 1064 nm light to a visible 532 nm beam and a mid-infrared beam ranging between 2200 and 4000 cm^–1^. SFG is achieved when the visible and infrared beams overlap spatially and temporally on the sample. The intensity of the sum frequency light as a function of IR frequency is a vibrational spectrum of the surface species measured in the visible region. All the spectra reported in this work were acquired with an SSP polarization combination, where the letters indicate the polarization of the sum frequency, visible, and IR beams. The CPD was measured by KPFM with an MFP-3D Asylum Research system, using conductive Pt/Ir tips located in the air side of the graphene electrode. The cantilever holding the tip was mechanically oscillated at its 75 kHz resonance frequency and simultaneous modulated with a 2 kHz AC bias of 3.5 V amplitude. Tip-sample CPD mapping was obtained in single-pass mode with side-band detection. For Raman spectroscopy, a laser beam with λ = 532 nm was focused on the graphene sample through a long working distance objective (Olympus, 100×, 0.5 NA) with a spatial resolution of 0.2 μm. The sample holder used for the Raman experiments is the same as that in reference (19).

## Supporting Information Available

(S1) Preparation of fresh graphene for SFVS, confocal Raman spectroscopy, and KPFM; (S2) Raman characterization of graphene; (S3) contamination detection in the CH region by SFVS and the C–C region by Raman spectroscopy; (S4) contamination detection in the dangling OH region by SFVS; (S5) Time evolution of the UV-treated graphene sample under ambient conditions; (S6) surface charge deduction from SF spectra in the bonded OH region; (S7) contact potential distribution for different biases by KPFM; and (S8) cyclic voltammetry for the graphene–electrolyte system (PDF)
