# Supplementary material for: Nature of the Electrical Double Layer on Suspended Graphene Electrodes
Source: J Am Chem Soc. 2022 Jul 18;144(29):13327–33. doi: 10.1021/jacs.2c03344 (PMC9335527; doi:10.1021/jacs.2c03344)
Supplement: Supplementary file 1 — ja2c03344_si_001.pdf [file ja2c03344_si_001.pdf]

Supplementary Information

The nature of the electrical double layer on suspended graphene electrodes

Shanshan Yang [1], Xiao Zhao [1,3], Yi-Hsien Lu [1], Edward S. Barnard [4], Peidong Yang [1,2], Artem Baskin [4,5], John W. Lawson [5], David Prendergast [4], Miquel Salmeron [1,3,*]

[1] Materials Sciences Division, Lawrence Berkeley National Laboratory, Berkeley, California 94720, USA.

[2] Department of Chemistry, University of California-Berkeley, Berkeley, California 94720, USA.

[3] Department of Materials Science and Engineering, University of California, Berkeley, California 94720, USA.

[4] Molecular Foundry, Lawrence Berkeley National Laboratory, Berkeley, California 94720, USA.

[5] NASA Ames Research Center, Moffett Field, California 94035, USA

S1. Preparation of fresh graphene for SFVS, confocal Raman spectroscopy and KPFM

S2. Raman characterization of graphene

S3. Contamination detection in CH region by SFVS and C-C region by Raman

S4. Contamination detection in dangling OH region by SFVS

S5. Time evolution of UV treated graphene sample under ambient condition

S6. Surface charge deduction from SF spectra in bonded OH region

S7. Contact potential distribution for different biases by KPFM

S.8 Cyclic Voltammetry for graphene-electrolyte system

S1. Preparation of fresh graphene for SFVS, confocal Raman spectroscopy and KPFM

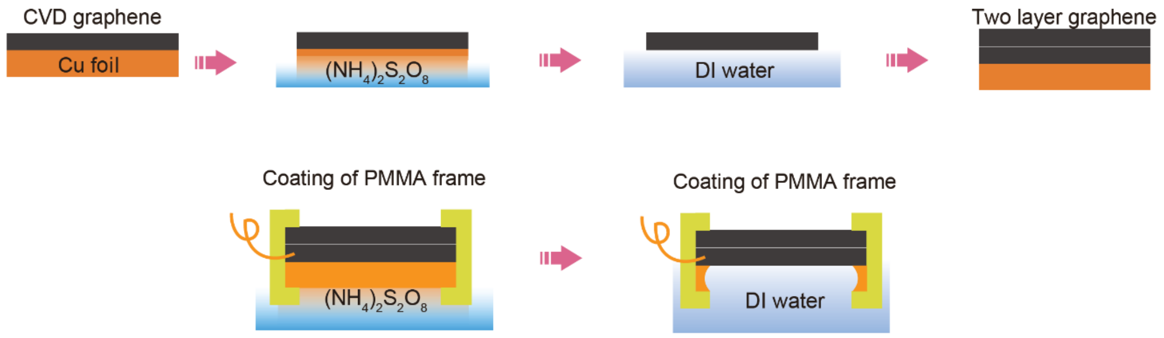

Figure S1 Schematic representation of the preparation of suspended graphene electrodes.

In constructing the suspended graphene electrode for SFVS and Raman, we adopted a 2-layer graphene structure, following the procedure described in Fig. S1. CVD-grown graphene on copper foil was first put on a 0.1 M $Na_2S_2O_8$ solution for 4 hours to etch away the copper beneath, after which the solution was changed to deionized (DI) water (18.2 MΩ) by syringe. The floating suspended single layer graphene was then scooped up by another CVD-grown graphene on copper and annealed at 120 °C for 1 hour to evaporate any residual water in-between so that the two layers of graphene stick together. A PMMA solution was then applied to the edges of graphene and copper to prevent the edges from dissolving into etching solution later. A copper wire was then attached to the copper foil through the PMMA to connect the graphene electrode to a potentiostat. Finally, the copper in the middle was etched away by 0.1 M $Na_2S_2O_8$ solution, leaving suspended graphene floating on DI water with a copper frame on the edge.

The details of graphene electrode fabrication for KPFM can be found in previous works[1]. In brief, commercial single-layer graphene grown on Cu foil (Graphene Factory) by chemical vapor deposition (CVD) was used as base material. Residual graphene on the other side of the foil was removed by O2 plasma. An adhesive Al foil frame window was stuck to the untreated side and then floated on a Cu etchant solution (~90ml 0.2 M sodium persulfate). After etching out the Cu the graphene with the Al window frame, was transferred to a deionized water reservoir (~500 ml) to remove salt residues. The transfer process to the water reservoir was repeated twice to ensure complete salt removal. A Cr (2.5 nm)/Au (25 nm) coated SiN membrane (100 nm thickness) perforated with an array of 1000 nm or 500 nm circular holes (Norcada) was placed in the reservoir underneath the graphene and carefully lifted up from the air/water interface to complete the graphene transfer. After this the membranes were annealed in vacuum (lower than 10-6 torr) up to 573K to increase the adhesion between graphene and Au.

S2. Raman characterization of graphene

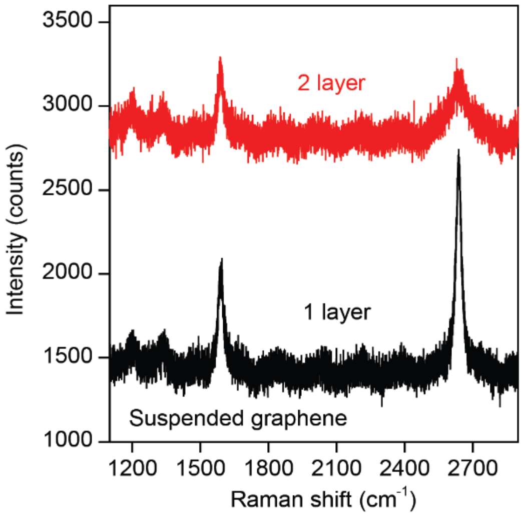

Figure S2. Raman spectra of 1-layer and 2-layer graphene.

Raman spectra of CVD-grown graphene were measured under ambient conditions using a 532 nm continuous laser. The intensity ratio of the G mode at 1587.3 cm^{-1} to the 2D mode at 2659 cm^{-1} is 0.53 for 1-layer graphene, indicating the good quality of the single layer graphene. The Raman spectra of 2-layer graphene is similar to that found in the literature[2], featuring the broadening of the 2D peak by increased channels for double resonance Raman process.

S3. Graphene contamination detection

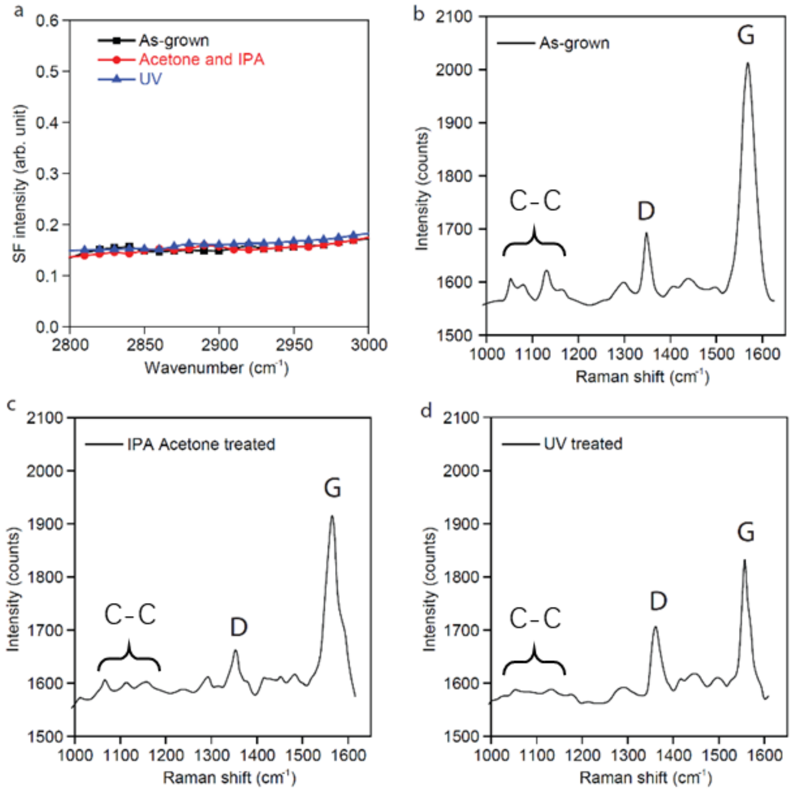

Figure S3. (a) SFVS spectra of suspended graphene on water in the CH stretching region after three cleaning treatments: IPA and acetone wash (red), UV treatment for 5 min (blue), and as grown, i.e., untreated (black). (b-d) Raman spectra following the same treatments.

To evaluate the degree of hydrocarbon contamination on graphene, we measured the CH stretching region in the SFVS. However, the signal is too small to analyze because of the disordered nature of the hydrocarbon layer which makes it undetectable due to the selection rules of SFVS. Raman spectra in Fig. S3 (b-c) however shows the peaks from C-C stretch at around 1100 cm^{-1}, CH_2 twist at around 1300 cm^{-1}, and CH_2 bending at around 1420 cm^{-1} which can be used to evaluate the amount of hydrocarbons present at the surface. By normalizing the above peaks to those of a sample prepared with a monolayer coverage of polyethylene, we could evaluate the surface contamination for the 3 sample as: 13.8 % of a monolayer, for the as-grown sample, 12.6 % for the IPA/ acetone washed sample, and (3) 5.1% for the UV-treated sample respectively.

S4. Effect of hydrocarbon contamination on the dangling OH stretch peak intensity by SFVS

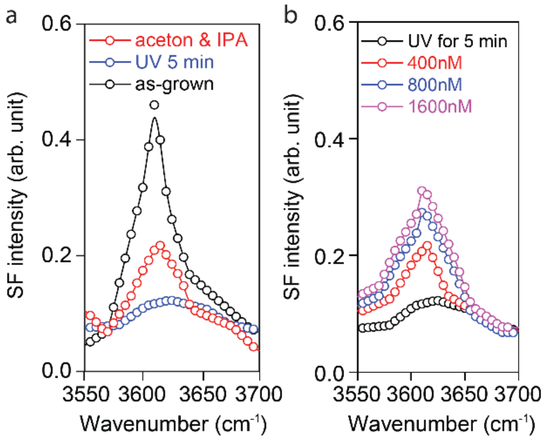

Figure S4 (a) SF spectra of the graphene-pure water interface in the dangling OH stretch region for untreated graphene (black), UV treated graphene for 5 min (blue), and graphene washed in an acetone-IPA solution (red). (b) SF spectra of the graphene water interface on a sample prepared by UV treatment followed by immersion in a polyethylene/CCl_4 solution of different concentrations up to 1.6M. The saturation of the dangling bond peak after immersion in a 1.6M polyethylene solution is assigned to the formation of a complete monolayer of polyethylene, which is used to calibrate the hydrocarbon contamination using the intensities of the corresponding Raman spectra peaks in Fig. S3.

It is well-known that in the SFVS peak intensity of the dangling OH bond stretch around 3620 cm^{-1} is a good indicator of the hydrophobicity of the interface.[3] Since a decreased intensity of the dangling OH bond stretch around 3620 cm^{-1} indicates that the surface is becoming more hydrophilic, it appears that washing in an acetone-isopropyl (IPA) solution improves hydrophilicity, but not sufficiently to remove all contaminants. The UV treatment is the most efficient because the dangling peak intensity is smallest after that treatment. The more intense dangling OH peak in the 'as received' samples indicates a strong hydrophobic character which is connected to the higher degree of hydrocarbon contamination, as often reported in experiments in ambient conditions.[4, 5] To quantify the degree of hydrocarbon contamination we immersed the graphene sample in a polyethylene/CCl_4 solution to produce a coverage of hydrocarbons that could serve as a reference for the degree of contamination.[4] The results, shown in Fig. S4b, indicate that the dangling OH peak in the SFVS increases and saturates when the polyethylene concentration in the solution reaches 1.6M. We assign this saturation to the completion of 1 monolayer (ML).

S5. Time evolution of UV treated graphene sample under ambient conditions

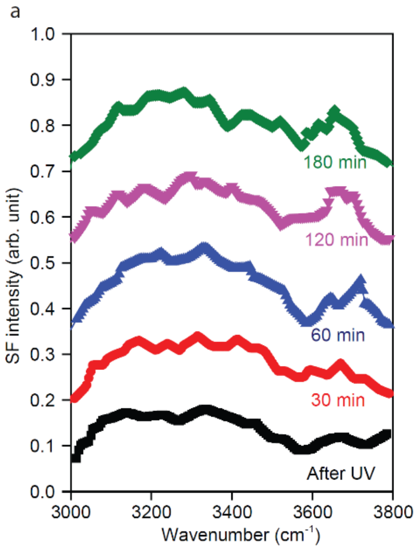

Figure S5. SFVS spectra of UV treated graphene on water under ambient conditions as a function of time.

Although the UV-treated graphene sample is the cleanest one among other treatments there are two difficulties using it as an electrode. The first one is that under ambient condition it is impossible, at least in our environment, to prevent the graphene sample from getting contaminated by air-born contaminants during the measurements, which normally requires several hours. As shown in Fig. S5, the SFVS of the UV cleaned sample changes with time, especially in the dangling OH region around 3620 cm^{-1}. As can be seen, the spectrum taken at very beginning is different from the ones taken 3 hours later, where the growth of the dangling OH bond peak is increasing. This time evolution of the spectra is observed in several repetitions, with some variability in the detailed shape and timing. A difficulty of a different nature is that the graphene becomes fragile after the UV treatment, probably because of oxidation with generated ozone. We therefore did not used the cleanest UV treated samples for this work, which in our laboratory conditions has a stable residual contamination level of 0.14% of a monolayer relative to the saturated polyethylene layer.

S6. Calculation of the surface charge from the intensity of the SFVS peaks in the bonded OH region

The measured SF intensity is proportional to $|\chi_{S,eff}^{(2)}(\omega)|^2$, where [6]

$$\chi_{S,eff}^{(2)}(\omega) = \chi_S^{(2)} + \int_{0^+}^{\infty} \vec{\chi}_B^{(3)} \cdot \hat{z}E_{DC}(z)e^{i\Delta k_z z}dz \tag{S1}$$

Here, $\chi_S^{(2)}$ and $\chi_B^{(3)}$ denote the surface nonlinear susceptibility and the third-order bulk nonlinear susceptibility of water, respectively. $\Delta k_z = k_{SF,z} + k_{VIS,z} + k_{IR,z}$ is the phase mismatch of the reflected SF process and $\hat{z}E_{DC}$ is the DC electric field in the electric double layer along the surface normal. For sufficiently low surface charge density (below 0.05 e/nm^2), the interfacial water structure is hardly perturbed, with $\chi_S^{(2)}$ essentially the same as that at the neutral suspended graphene/water interface, which can be measured from the CNP spectra of the salt solution. [6]

Thus, the spectral change due to surface charges, given by

$$\Delta\chi_{S,eff}^{(2)}(\omega) = \chi_{S,eff}^{(2)}(\omega) - \chi_S^{(2)}(\omega) = \chi_B^{(3)}(\omega)\int_{0^+}^{\infty} E_{DC}(z)e^{i\Delta k_z z}dz, \tag{S2}$$

can be obtained from measured spectra.

The electric field distribution $E_{DC}(z)$ in the EDL is related to the surface charge density (σ) by the Gouy Chapman model. For an electrolyte solution, [7]

$$E_{DC}(\mathrm{z}) = \frac{4k_BT}{e}\frac{\kappa\tanh(\frac{e\phi_0}{4k_BT})}{\tanh^2(\frac{e\phi_0}{4k_BT})\exp(-2\kappa z)-1}\exp(-\kappa z) \tag{S3}$$

with

$$\kappa = \frac{1}{\lambda_D} = (\frac{2ce^2}{\varepsilon_0\varepsilon_r k_B T})^{1/2} \tag{S4}$$

and

$$\phi_0 = -\frac{2k_BT}{e}\sinh^{-1}(\frac{e\sigma}{(8k_BT\varepsilon_0\varepsilon_r c)^{1/2}}) \tag{S5}$$

Here, λ_D is the Debye length, c and ε_r are the ionic strength and the relative dielectric constant of the solvent, respectively.

Since $\chi_B^{(3)}$ for water is known from an early measurement [6] and the ionic strength c known from each salt solution, σ can be found by fitting the measured $\Delta\chi_{S,eff}^{(2)}(\omega)$ spectrum for each potential using Eq. S2-S5. The result is shown in Fig. 4c in the main text.

S7. Contact potential distribution for different biases by KPFM

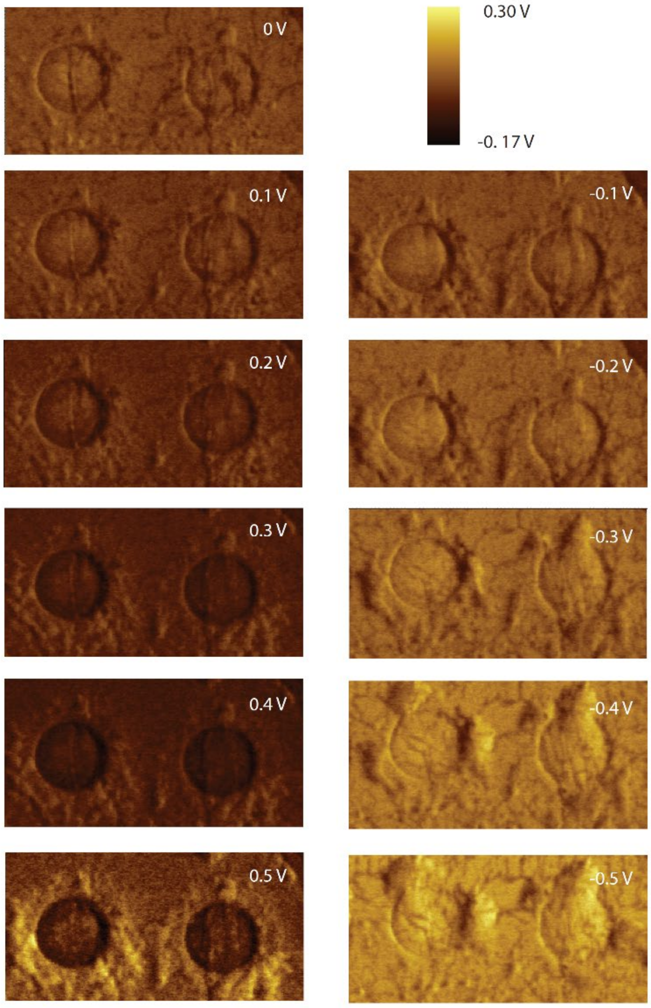

Figure S6. Contact Potential Difference (CPD) images from the KPFM measurements on suspended graphene over micrometer diameter holes in a 100 nm thick SiN membrane covered with a 20 nm gold layer. The bias on the graphene varied from -0.5 V to +0.5 V relative to the Pt counter electrode. Images at -04V and +0.4V are used in Fig. 4b of the main text.

S.8 Cyclic Voltammetry for graphene-electrolyte system

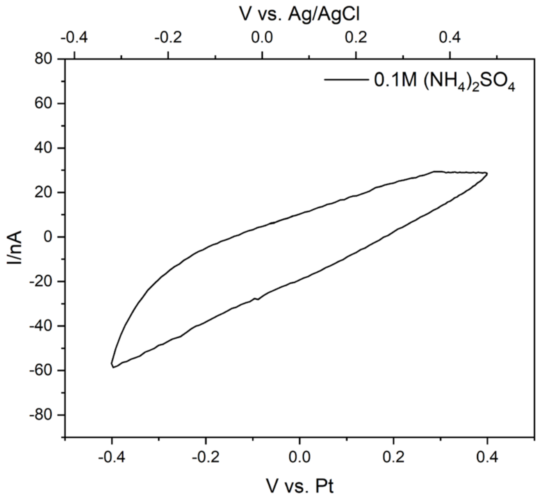

Figure S7. Representative Cyclic Voltammetry curve for graphene in contact with 0.1M $(NH_4)_2SO_4$ electrolyte. Other electrolytes will be very similar.

Reference

(1) Lu, Y.-H.; Morales, C.; Zhao, X.; Van Spronsen, M. A.; Baskin, A.; Prendergast, D.; Yang, P.; Bechtel, H. A.; Barnard, E. S.; Ogletree, D. F.; et al. Ultra-thin Free-Standing Oxide Membranes for Electron and Photon Spectroscopy Studies of Solid-gas and Solid-liquid Interfaces. *Nano Lett* **2020**. DOI: 10.1021/acs.nanolett.0c01801. Lu, Y.-H.; Larson, J. M.; Baskin, A.; Zhao, X.; Ashby, P. D.; Prendergast, D.; Bechtel, H. A.; Kostecki, R.; Salmeron, M. B. Infrared Nanospectroscopy at the Graphene-Electrolyte Interface. *Nano Lett* **2019**.

(2) Malard, L. M.; Pimenta, M. A.; Dresselhaus, G.; Dresselhaus, M. S. Raman spectroscopy in graphene. *Phys. Rep.* **2009**, *473* (5), 51-87. DOI: https://doi.org/10.1016/j.physrep.2009.02.003.

(3) Tian, C. S.; Shen, Y. R. Structure and charging of hydrophobic material/water interfaces studied by phase-sensitive sum-frequency vibrational spectroscopy. *Proc Natl Acad Sci U S A* **2009**, *106* (36), 15148-15153. DOI: 10.1073/pnas.0901480106 PubMed.

(4) Su, Y.; Han, H.-L.; Cai, Q.; Wu, Q.; Xie, M.; Chen, D.; Geng, B.; Zhang, Y.; Wang, F.; Shen, Y. Polymer adsorption on graphite and CVD graphene surfaces studied by surface-specific vibrational spectroscopy. *Nano letters* **2015**, *15* (10), 6501-6505.

(5) Temiryazev, A.; Frolov, A.; Temiryazeva, M. Atomic-force microscopy study of self-assembled atmospheric contamination on graphene and graphite surfaces. *Carbon* **2019**, *143*, 30-37. DOI: https://doi.org/10.1016/j.carbon.2018.10.094.

(6) Wen, Y. C.; Zha, S.; Liu, X.; Yang, S.; Guo, P.; Shi, G.; Fang, H.; Shen, Y. R.; Tian, C. Unveiling Microscopic Structures of Charged Water Interfaces by Surface-Specific Vibrational Spectroscopy. *Phys Rev Lett* **2016**, *116* (1), 016101. DOI: 10.1103/PhysRevLett.116.016101.

(7) Adamson, A. W.; Gast, A. P. *Physical Chemistry of Surfaces*; John Wiley & Sons, Inc., 1997.
